# Mesenchymal stromal cell-derived extracellular vesicles attenuate lung ischemia-reperfusion injury and enhance reconditioning of donor lungs after circulatory death

**DOI:** 10.1186/s12931-017-0704-9

**Published:** 2017-12-21

**Authors:** Matthew L. Stone, Yunge Zhao, J. Robert Smith, Mark L. Weiss, Irving L. Kron, Victor E. Laubach, Ashish K. Sharma

**Affiliations:** 1 Department of Surgery, University of Virginia, P.O. Box 801359, Charlottesville, VA 22908 USA; 2 Department of Anatomy and Physiology, Kansas State University, Manhattan, KS USA

**Keywords:** Mesenchymal stromal cells, Microvesicles, Ischemia-reperfusion injury, Ex vivo lung perfusion, Donation after circulatory death

## Abstract

**Background:**

Lung ischemia-reperfusion (IR) injury after transplantation as well as acute shortage of suitable donor lungs are two critical issues impacting lung transplant patients. This study investigates the anti-inflammatory and immunomodulatory role of human mesenchymal stromal cells (MSCs) and MSC-derived extracellular vesicles (EVs) to attenuate lung IR injury and improve of ex-vivo lung perfusion (EVLP)-mediated rehabilitation in donation after circulatory death (DCD) lungs.

**Methods:**

C57BL/6 wild-type (WT) mice underwent sham surgery or lung IR using an in vivo hilar-ligation model with or without MSCs or EVs. In vitro studies used primary iNKT cells and macrophages (MH-S cells) were exposed to hypoxia/reoxygenation with/without co-cultures with MSCs or EVs. Also, separate groups of WT mice underwent euthanasia and 1 h of warm ischemia and stored at 4 **°**C for 1 h followed by 1 h of normothermic EVLP using Steen solution or Steen solution containing MSCs or EVs.

**Results:**

Lungs from MSCs or EV-treated mice had significant attenuation of lung dysfunction and injury (decreased edema, neutrophil infiltration and myeloperoxidase levels) compared to IR alone. A significant decrease in proinflammatory cytokines (IL-17, TNF-α, CXCL1 and HMGB1) and upregulation of keratinocyte growth factor, prostaglandin E2 and IL-10 occurred in the BAL fluid from MSC or EV-treated mice after IR compared to IR alone. Furthermore, MSCs or EVs significantly downregulated iNKT cell-produced IL-17 and macrophage-produced HMGB1 and TNF-α after hypoxia/reoxygenation. Finally, EVLP of DCD lungs with Steen solution including MSCs or EVs provided significantly enhanced protection versus Steen solution alone. Co-cultures of MSCs or EVs with lung endothelial cells prevents neutrophil transendothelial migration after exposure to hypoxia/reoxygenation and TNF-α/HMGB1 cytomix.

**Conclusions:**

These results suggest that MSC-derived EVs can attenuate lung inflammation and injury after IR as well as enhance EVLP-mediated reconditioning of donor lungs. The therapeutic benefits of EVs are in part mediated through anti-inflammatory promoting mechanisms via attenuation of immune cell activation as well as prevention of endothelial barrier integrity to prevent lung edema. Therefore, MSC-derived EVs offer a potential therapeutic strategy to treat post-transplant IR injury as well as rehabilitation of DCD lungs.

## Background

Lung transplantation provides a curative hope for many with end-stage pulmonary disease but the long-term survival and outcome remain the poorest of any solid organ transplant with survival estimates demonstrating approximately 50% mortality after 5-years post-transplant [1]. One of the major complications is lung ischemia-reperfusion (IR) injury following transplantation which imposes a significant threat to graft and recipient survival thereby causing primary graft dysfunction [2]. Lung IR injury involves oxidative stress and crosstalk between many cell types including T cells, macrophages and alveolar type II epithelial cells. Recent studies from our group have shown that iNKT cell-produced IL-17 is critical for the initiation and progression of lung IR injury [3]. We have previously demonstrated that macrophage produced-HMGB1 (high mobility group box 1) can activate RAGE (receptor for advanced glycation end-products) on iNKT cells to amplify IL-17 production to mediate lung IR injury [4]. However, pharmacological modalities to immunomodulate the activation of these critical immune cells responsible for initiating lung IR injury remain elusive. Therefore, the first aim of this study was to investigate the anti-inflammatory and immunomodulatory role of human umbilical cord-derived mesenchymal stromal cells (MSCs) and MSC-derived extracellular vesicles (EVs) to attenuate lung injury and inflammation after IR.

Recent studies have shown that MSCs as well as MSC-derived EVs have the potential to mitigate lung injury and inflammation in various disease models [5–8]. EVs released by MSCs include apoptotic bodies, exosomes or microvesicles (MVs) [9]. The apoptotic bodies (>1000 nm) are products of dying cells, while exosomes (20–100 nm) have endosomal biogenesis and can be composed of lipids, proteins, and nucleic acids [10]. MVs (100–1000 nm) are generated by budding off from the plasma membrane and can contain cellular fractions consisting of microRNAs, mRNAs, proteins and mitochondria. Both exosomes and MVs can interact with other cells via paracrine secretions or internalized by cell-cell interactions through ligand-receptor pathways leading to biologic responses. Therefore, our aim was to investigate the immunomodulatory potential of MSC-derived EVs in the attenuation of inflammation and dysfunction associated with lung IR injury.

Furthermore, hypothermic organ storage is associated with oxidative stress, sodium pump inactivation, intracellular calcium overload, iron release, and cell death that induce cell surface expression patterns and pro-inflammatory mediators for leukocyte activation during the reperfusion period [11]. Additionally, we and others have previously shown that post-transplant lung function can be significantly improved by ex-vivo lung perfusion (EVLP) with Steen solution in non-heart beating donor lungs with warm ischemia [12–15]. In this study, we hypothesize that the protective effects of EVLP can be further enhanced by treatment with MSCs or EVs during lung preservation leading to enhancement of endothelial cell barrier integrity in donor lungs.

## Methods

### Animals

This study utilized 8–12 week old male C57BL/6 wild-type mice (Jackson Laboratory, Bar Harbor, ME) which were randomly assigned to different groups that underwent either the hilar ligation model of lung IR or DCD followed by EVLP. This study conformed to the National Institutes of Health guidelines and was conducted under animal protocols approved by the University of Virginia’s Institutional Animal Care and Use Committee.

### Lung IR model

An in vivo hilar ligation model of lung IR was used wherein mice undergoing IR were subjected to 1 h left lung ischemia (via left hilar occlusion) followed by 2 h of reperfusion as previously described [3, 16]. Mice were treated with or without MSCs or EVs (1 × 10^6^) given intratracheally 1 h prior to ischemia. Sham animals received the same surgery but without hilar occlusion. Mice were anesthetized with inhaled isoflurane, intubated with PE-60 tubing and connected to a pressure-controlled ventilator (Harvard Apparatus Co, South Natick, MA). Mechanical ventilation with room air was performed at 150 strokes/min, 0.5 cm^3^ stroke volume, and peak inspiratory pressure < 20 cm H_2_O. Heparin (20 U/kg) was given immediately preceding the ischemic period via external jugular injection to minimize thrombosis in the pulmonary vasculature during ischemia. Reperfusion was achieved by removing the clip, tube and hilar suture. The mouse was extubated and placed back in the cage during the 2-h reperfusion period. To minimize pain and discomfort, an analgesic (0.2 mg/kg buprenorphine) was administered to all animals at the beginning of surgical intervention.

### Murine lung DCD and EVLP

A murine lung DCD and EVLP model was used as previously described [17]. Mice were anesthetized and euthanized by cervical dislocation followed by a 60-min period of “no-touch” warm ischemia. The left atrium was then vented via an atriotomy followed by infusion of the lungs with 3 mL 4 °C Perfadex® solution (Vitrolife Inc., Denver, CO) supplemented with THAM Solution (Vitrolife, Kungsbacka, Sweden). The chest was then packed with ice and the lungs underwent cold static preservation for 60 min at 4 °C followed by EVLP using either KH (Krebs Henseleit) buffer, Steen solution, or Steen solution supplemented with MSCs or EVs (3 × 10^6^). The lungs were perfused with Steen solution (EXVIVO Perfusion Inc., Englewood CO) at a constant rate of 60 μl/g body weight/min. Steen solution within the circuit was gradually warmed from 4 °C to 37 °C (over approximately 10 min), and EVLP continued for 60 min. Steen solution was supplemented with 10,000 IU heparin, 500 mg cefazolin and 500 mg methylprednisolone per 1500 mL, modeling preclinical and clinical EVLP protocols (10, 12).

### Pulmonary function

Pulmonary function was evaluated using an isolated, buffer-perfused mouse lung system (Hugo Sachs Elektronik, March-Huggstetten, Germany) as previously described [17, 18]. Briefly, mice were anesthetized with ketamine and xylazine and a tracheostomy was performed, and animals were ventilated with room air at 100 breaths/min at a tidal volume of 7 μl/g body weight with a positive end expiratory pressure of 2 cm H_2_O using a MINIVENT mouse ventilator (Hugo Sachs Elektronik, March-Huggstetten, Germany). The lungs were perfused at a constant flow of 60 μl/g body wt/min with KH buffer (Sigma-Aldrich, St. Louis, MO) containing 0.1% glucose and 0.3% HEPES (335–340 mOsmol/kg H_2_O). The buffered perfusate and isolated lungs were maintained at 37 °C throughout the experiment by use of a circulating water bath. The lungs were maintained on the system for a 5-min equilibration period before data was recorded for an additional 5 min.

### Human Mesenchymal Stromal cell isolation and characterization

Human umbilical cord-derived MSCs were isolated from Wharton’s jelly and characterized by flow cytometry confirming a pattern consistent with MSC population showing an expression of CD90, CD73, CD105 and CD44 [19, 20].The cords were discarded tissues from apparently healthy, anonymous donors. The work with human tissues was reviewed by the Kansas State University Institutional Human Subjects Review Board and deemed to be not human subject’s research (IRB review #5189). MSCs lacked expression of CD45, CD34, CD11b, CD19, and HLA-DR. MSCs were differentiated with StemPro differentiation kits for chondrogenesis, adipogenesis and osteogenesis following the protocols included with the kits (Life Technologies, Grand Island, NY), as previously reported [21].

### Isolation and characterization of EVs

EVs were obtained from supernatants of MSCs cultured overnight in RPMI deprived of Fetal Bovine Serum (FBS) and supplemented with 0.5% of BSA (Sigma Aldrich, St. Louis, MO). The cell viability was 99% for MSCs as detected by trypan blue exclusion. To obtain EVs, the supernatants from MSCs underwent centrifugation at 10,000 g for 20 min to remove debris, and then cell-free supernatant were centrifuged at 100,000 g (Beckman Coulter Optima ultracentrifuge) for 1 h at 4 °C, washed in serum-free medium HEPES (Sigma) and subjected to a second ultracentrifugation under similar conditions. The mean size and particle concentration was evaluated using Nanosight LM10 instrument (Malvern Instruments, Worcestershire, UK). MSCs were used to successfully isolate and characterize EVs which were isolated from the culture media. The mean size (164 ± 10.4 nm) and particle concentration of EVs were calculated by the Nanoparticle Tracking Analysis software. Further characterization of EVs was performed by imaging flow cytometry (ImageStreamX imaging FC (ISX) [22] (EMD Millipore, Billerica, MA) using CD90-FITC, CD44 APC, CD73-PerCP-Cy5.5 (eBioscience, Waltham, MA) and a lipophilic dye (DilC_18_; Molecular Probes, Eugene, OR). Imaging flow cytometry analysis of EVs by ImageStream confirmed the cell surface marker expression for MSCs (i.e. CD90, CD44 and CD73) thereby confirming the source of origin of the EVs. The protein content of EVs was quantified by Bradford method (BioRad, Hercules, CA). Total RNA was isolated from EVs using the Qiagen RNAeasy kit (Qiagen Inc., Valencia, CA) and quality and concentration was assessed by NanoDrop UV spectrophotometer (NanoDrop Technologies, Wilmington, DE). Protein and total RNA quantities of 50 μl EVs were 54.34 ± 5.99 μg and 35.97 ± 5.4 ng, respectively, released by 5 × 10^6^ cultured MSCs. This protein concentration falls in the range of previously reported studies using EVs in lung and kidney injury disease models [23, 24].

### Bronchoalveolar lavage

At the conclusion of pulmonary function measurements, left lungs were lavaged with 0.4 ml phosphate buffered saline. The BAL fluid was centrifuged at 1500 rpm for 10 mins 4 °C, and the supernatant was stored at −80 °C.

### Cytokine analysis

Cytokine concentrations in BAL fluid were quantified using the Bioplex Bead Array technique and a multiplex cytokine panel assay (Bio-Rad Laboratories, Hercules, CA) as previously described [3].

### Myeloperoxidase (MPO) measurement

MPO levels were measured in BAL fluid using a mouse MPO ELISA kit (Hycult Biotech, Uden, The Netherlands). MPO is abundant in the azurophilic granules of polymorphonuclear neutrophils and was used as an indicator of neutrophil activation and infiltration into alveolar airspaces.

### Lung wet/dry weight

Lungs were weighed and then desiccated until a stable dry weight was achieved. Lung wet/dry weight ratio was then calculated as an indicator of edema. Separate groups of animals that did not undergo BAL were used to measure lung wet/dry weight.

### Immunohistochemistry

Immunostaining to identify neutrophils was performed as described previously [3, 4, 16]. Lungs were inflation-fixed at 20 cm H_2_O with 4% paraformaldehyde and paraffin embedded. Immunostaining of lung sections was performed with rat anti-mouse neutrophil antibody (LY6B.2, Bio-Rad Laboratories) using Vectastain ABC kit (Vector Laboratories, Burlingame, CA). Purified normal rat immunoglobulin G (eBioscience Inc., San Diego, CA) was used as a negative control. Alkaline phosphatase–conjugated anti-rat immunoglobulin G (Sigma Aldrich, St. Louis, MO) was used as the secondary antibody, and signals were detected with Fast-Red (Sigma Aldrich). Sections were counterstained with hematoxylin. For each lung section, the number of neutrophils per high power field were counted by a blinded reviewer in five random fields at 40X magnification and averaged per tissue sample. These counts did not distinguish between cells in various compartments of the lung (e.g. airspace, interstitial or vascular) but included all cells in peripheral lung tissue.

### In vitro hypoxia-reoxygenation (HR)

Primary murine iNKT cells (isolated as previously described [3, 16]) and MH-S macrophages were cultured overnight with/without MSCs or EVs in RPMI media containing 10% fetal bovine serum and 1% penicillin/streptomycin (Invitrogen, Carlsbad, CA) at 37 °C and 5% CO_2_. For exposure to HR, 24-well culture plates were placed in a humidified, sealed hypoxic chamber (Billups-Rothenberg, Del Mar, CA) that was purged with 95% N_2_ and 5% CO_2_ for 25 min to establish hypoxia as described previously [25]. The chamber was then placed in a cell culture incubator for 3 h after which it was opened and the culture media was immediately analyzed for O_2_concentration using a blood-gas analyzer (Chiron Diagnostics). The partial percentage of O_2_ in the culture media after hypoxia exposure was consistently found to be 5% versus 21% in normoxic cultures. Reoxygenation was achieved by removing the plates from the hypoxic chamber and placing them in a normoxic, humidified incubator (37 °C, 5% CO_2_) for 1 h.

### Transendothelial migration assay

Mouse primary lung microvascular endothelial cells (LMVECs) were cultured overnight in endothelial cell medium with supplements (Cell Biologics, Chicago, IL). LMVECs (10^5^ cells) were co-cultured on transwell membrane inserts with/without MSCs or EVs (0.5 × 10^5^) and exposed to hypoxia/reoxygenation for 4 h to perform the neutrophil transmigration assay per the manufacturer’s instructions (Cell Biolabs, Inc., San Diego, CA). Polymorphonuclear leukocytes (PMNs) were harvested from mouse spleens and isolated using a cell isolation kit (Miltenyi Biotec, Germany). PMNs were labeled with a fluorescent LeukoTracker dye and incubated with LMVECs for 4 h. The migratory PMNs at the bottom of the plate were then counted using a fluorescent plate reader at 480 nm/520 nm.

### Statistical analysis

All statistical analyses were performed using GraphPad Prism 6.0 software, and data are presented as the mean ± standard error of the mean. One-way ANOVA with post-hoc Tukey’s test or Student’s t-test were used as appropriate to compare experimental groups. Statistical significance was set at *P* < 0.05.

## Results

### Pulmonary dysfunction after IR is attenuated by MSC-derived EVs

**Fig. 1:**
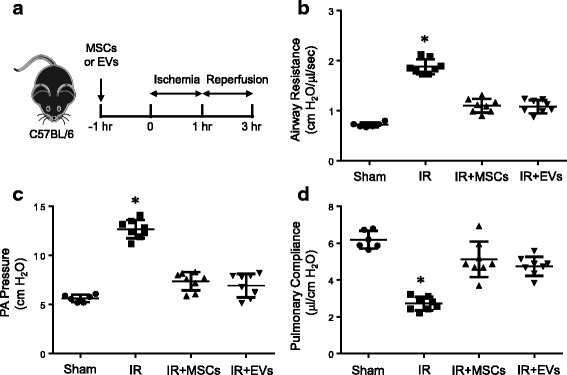
Pulmonary dysfunction after IR is attenuated by MSCs and EVs. **a** Schematic of murine lung IR protocol where pulmonary function was measured in WT mice after sham surgery or IR. **b-d** Significant lung dysfunction occurred after IR as demonstrated by increased airway resistance and pulmonary artery (PA) pressure as well as decreased pulmonary compliance compared to sham controls. Pretreatment with MSCs or EVs resulted in significantly reduced lung dysfunction after IR. *n* = 6–8/group; *p < 0.05 vs. all

The in vivo hilar ligation model of lung IR was used where WT mice were subjected to 1 h ischemia followed by 2 h of reperfusion with or without MSC or EV treatment (Fig. 1a). Significant pulmonary dysfunction occurred after IR in WT mice compared to sham as indicated by increased airway resistance (1.87 ± 0.05 vs. 0.72 ± 0.02 cm H_2_O/μl/s) and pulmonary artery pressure (12.6 ± 0.32 vs. 5.6 ± 0.15 cm H_2_O) as well as decreased pulmonary compliance (2.72 ± 0.13 vs. 6.2 ± 0.19 μl/cm H_2_O) (Fig. 1b-d). Lungs of mice treated with MSCs or MSC-derived EVs were protected after IR compared to untreated mice as shown by significantly decreased airway resistance (1.1 ± 0.04 and 1.08 ± 0.04 cm H_2_O/μl/s) and pulmonary artery pressure (7.3 ± 0.32 and 6.9 ± 0.42 cm H_2_O) as well as increased pulmonary compliance (5.1 ± 0.34 and 4.7 ± 0.18 μl/cm H_2_O). There was no significant difference in protection offered between MSCs and EVs after IR. Also, sham mice treated with MSCs or EVs did not change the lung function parameters compared to untreated shams (data not shown). These results show that MSC-derived EVs can mitigate lung dysfunction after IR and are comparably protective as MSCs themselves.

### Lung injury and inflammation after IR is attenuated by treatment with MSC-derived EVs

**Fig. 2:**
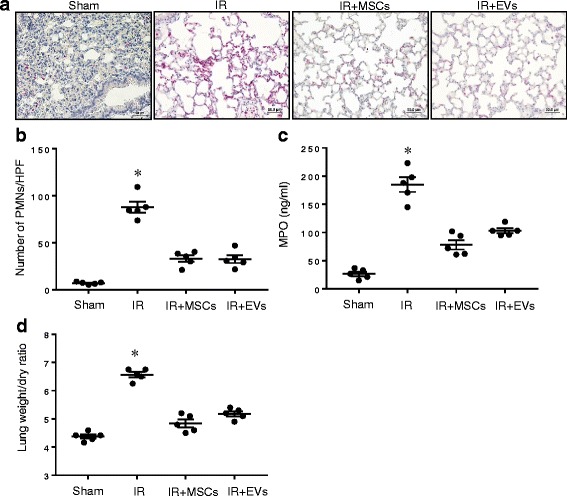
MSCs or EVs decrease neutrophil infiltration and activation as well as lung edema after IR**. a** Representative images showing neutrophil immunostaining in lung sections. Neutrophils are stained red and sections are counterstained with hematoxylin. Scale bars indicate 50 μm. **b** The number of neutrophils per high power field (HPF) was quantified from immunostained sections. Neutrophil infiltration was significantly attenuated after IR in mice treated with MSCs or EVs compared to untreated mice. **c** Myeloperoxidase (MPO) levels in bronchoalveolar lavage fluid was significantly decreased after IR in mice treated with MSCs or EVs. **d** Pulmonary edema (lung wet/dry weight) was significantly decreased after IR in wild-type mice treated with MSCs or EVs compared to untreated mice*. n* = 5/group; *p < 0.05 vs. all

**Fig. 3:**
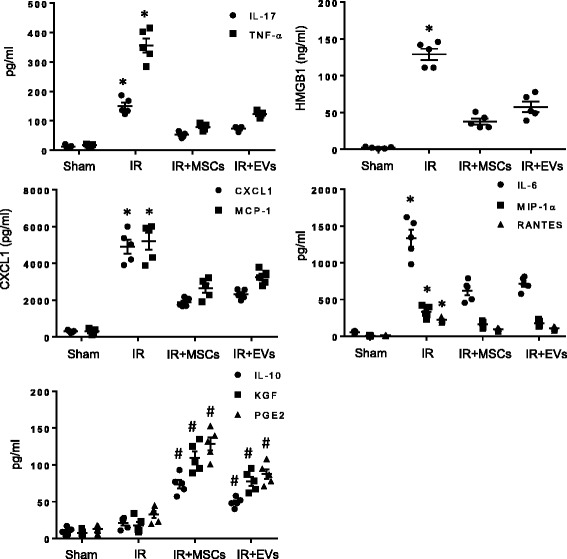
Lung inflammation after IR is attenuated by MSCs or EVs. Proinflammatory cytokine levels (IL-17, TNF-α, HMGB1, CXCL1, MCP-1, IL-6, MIP-1α and RANTES) were significantly attenuated in BAL fluid after IR in mice treated with MSCs or EVs versus IR alone. Anti-inflammatory cytokine (IL-10) expression as well as keratinocyte growth factor (KGF) and prostaglandin E2 (PGE2) levels were significantly increased in BAL fluid after treatment with MSCs or EVs after IR compared to IR alone. n = 5/group; *p < 0.05 vs. all; #*p* < 0.05 vs. IR

To determine the protective role of EVs on lung injury after IR, neutrophil infiltration in lung tissue and myeloperoxidase (MPO) levels in bronchoalveolar lavage fluid were measured (Fig. 2a-c). A marked increase in neutrophil infiltration and MPO levels occurred in WT mice after IR which was blocked by treatment with MSCs or EVs after IR compared to untreated mice. Similarly, pulmonary edema (wet/dry weight ratio), and pro-inflammatory cytokine expression were measured to evaluate the effect of MSC and EV-mediated effects on lung edema and inflammation, respectively. We observed a significant decrease in lung edema (Fig. 2d) and proinflammatory cytokine (IL-17, TNF-α, HMGB1, CXCL1, MCP-1, IL-6, MIP-1α, RANTES) production after treatment with MSCs or EVs in WT mice undergoing IR compared to IR alone (Fig. 3). Furthermore, expression of keratinocyte growth factor (KGF), prostaglandin E2 (PGE2) and IL-10 was increased in the BAL fluid of mice treated with MSCs or EVs after IR compared to IR alone (Fig. 3). These results confirm that MSCs or EVs can comparably and effectively attenuate lung inflammation, edema and neutrophil infiltration and activation after IR.

### MSCs or MSC-derived EVs attenuate hypoxia/reoxygenation-induced activation of iNKT cells and macrophages

**Fig. 4:**
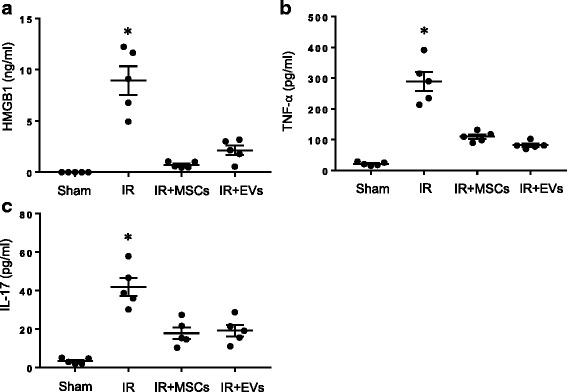
MSCs and EVs inhibit macrophage and iNKT cell activation after HR. **a-b** MH-S (alveolar macrophages) cells were exposed to hypoxia/reoxygenation (HR) and demonstrated a significant increase in HMGB1 and TNF-α production compared to normoxia (Norm) which was significantly attenuated by treatment with MSCs or EVs. **c** Similarly, HR-exposed iNKT cells produced increased levels of IL-17 compared to normoxia which was significantly attenuated by treatment with MSCs or EVs. n = 5/group; *, p < 0.05 vs. all

Since our previous studies have shown that macrophage-produced HMGB1 and TNF-α as well as iNKT cell-dependent IL-17 production mediate lung IR injury [3, 4, 25], we investigated the role of MSC-derived EVs in the mitigation of these pro-inflammatory mediators using hypoxia/reoxygenation (HR) as an in vitro surrogate model of IR. HR significantly increased the expression of HMGB1 and TNF-α in MH-S cells which were markedly decreased by co-culturing these cells with either MSCs or EVs (Fig. 4a-b). Similarly, HR-exposed iNKT cells induced a significant increase in IL-17 production which was inhibited by co-culturing iNKT cells with either MSCs or EVs (Fig. 4c). These results signify that MSCs and EVs can effectively inhibit immune cell activation and inflammation during lung IR and demonstrate a direct immunomodulatory function of MSCs and EVs.

### Msc-derived EVs improve lung function and reduces edema during EVLP of DCD lungs

**Fig. 5:**
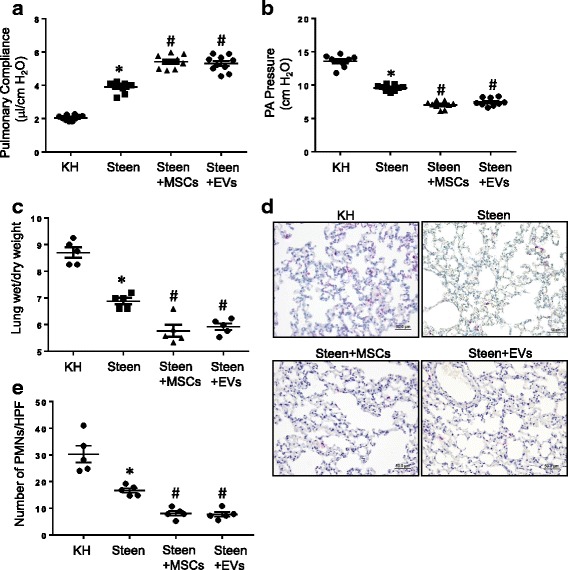
EVLP-directed delivery of MSCs or EVs enhances lung function and inhibits edema in DCD lungs. **a-b** EVLP with Steen solution significantly increased pulmonary compliance and reduced pulmonary artery (PA) pressure compared to EVLP with Krebs Henseleit (KH) buffer. Supplementation of Steen solution with MSCs or EVs provided enhanced improvement in lung function after EVLP compared to Steen solution alone. **c** Pulmonary edema was significantly decreased with MSC or EV-treatment during EVLP with Steen solution compared to EVLP with Steen solution alone. **d** Representative images showing neutrophil immunostaining in lung sections. Neutrophils are stained red and sections are counterstained with hematoxylin. **e** The number of neutrophils per high power field (HPF) was quantified from immunostained sections. Neutrophil infiltration was significantly attenuated after EVLP with Steen solution compared to KH. Supplementation of Steen solution with MSCs or EVs further decreased neutrophil infiltration compared to Steen solution alone. *n* = 5–10/group; *, *p* < 0.05 vs. KH; #*p* < 0.05 vs. Steen

To investigate the rehabilitative effects of EVs, we used a murine EVLP model as previously described [17]. Murine lungs after DCD underwent EVLP with Steen solution and demonstrated significantly increased pulmonary compliance and decreased pulmonary artery pressure versus EVLP with a control KH solution (Fig. 5a-b). Furthermore, EVLP with Steen solution supplemented with MSCs or EVs significantly improved pulmonary compliance and pulmonary artery pressure when compared to EVLP with Steen solution alone. In addition, EVLP with Steen solution significantly reduced pulmonary edema compared to KH buffer (wet/dry weight) (Fig. 5c). A significant decrease in neutrophil infiltration was observed in mouse lungs treated after EVLP with Steen solution compared to KH buffer (Fig. 5d-e). Moreover, supplementation of Steen solution with either MSCs or EVs further ameliorated neutrophil infiltration in the lungs compared to Steen solution alone. Therefore, in synergy with functional improvement, EVLP with Steen solution supplemented with MSCs or EVs also significantly reduced lung injury and edema compared to Steen solution alone.

### Msc-derived EVs inhibit neutrophil transmigration in endothelial cells after hypoxia-reoxygenation

**Fig. 6:**
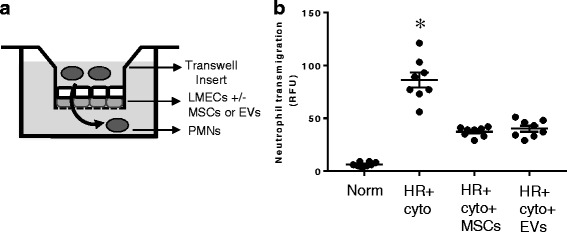
MSCs and EVs attenuate neutrophil transendothelial migration in lung microvascular endothelial cells (LMECs). **a** Schematic showing the transwell cell culture model used to assess neutrophil transendothelial migration. Primary murine neutrophils were added to LMEC monolayer co-cultured with or without MSCs or EVs. **b** Exposure of LMECs to HR and cytomix (cyto; HMGB1 and TNF-α; 50 ng/ml each) for 4 h showed a significant increase in neutrophil transmigration, which was significantly attenuated by co-cultures with MSCs or EVs. *n* = 8/group; *, *p* < 0.05 vs. all

To further investigate the protective role of MSC-derived EVs in protection against lung edema, primary lung microvascular endothelial cells (LMECs) were exposed to HR plus cytomix (TNF-α and HMGB1) and neutrophil transmigration through the endothelial monolayer was assessed (Fig. 6a). A significant increase in neutrophil transendothelial migration occurred in LMECs alone after exposure to HR and cytomix which was significantly attenuated by inclusion of MSCs or EVs which were co-cultured with LMECs (Fig. 6b). These results confirm that neutrophil transendothelial migration in lung ECs can be effectively blocked by EVs to protect the endothelial barrier integrity against pulmonary edema, which is a hallmark of injury in DCD lungs as well as after IR.

## Discussion

In this study, our primary goal was to elucidate the protective immunomodulatory properties of MSC-derived EVs in the mitigation of lung IR injury as well as enhancement of EVLP-mediated reconditioning of DCD lungs. We observed that EVs offered significant protection from lung dysfunction, inflammation, edema and neutrophil infiltration after IR. Furthermore, in vitro studies demonstrated that EVs can attenuate pro-inflammatory secretion of HMGB1, TNF-α from macrophages and IL-17 production from iNKT cells thereby demonstrating the direct immunomodulatory capacities of EVs in immune cell activation during lung IR injury. Finally, our results indicate that supplementation of Steen solution with EVs during EVLP enhances the protective and rehabilitative effects of EVLP which is likely due to the protective effects of EVs against loss of endothelial barrier integrity.

Despite the favorable safety profile from clinical trials, MSCs may have the capacity for spontaneous malignant transformation depending on the in vitro preparation of the cells [26–28]. Therefore, a potential therapy that can harness the beneficial aspects of MSCs would represent the next therapeutic avenue for the clinical application. MSC-derived EVs are small vesicles (100–1000 nm) without nuclei released by the cell, and can offer a viable cell-free approach to target inflamed and injured tissues. More importantly, EVs can be as biologically active as the stem cells themselves in vitro as well as in vivo [23, 29–32]. However, the exact role of EVs in the modulation of immune cell activation and mitigation of lung IR injury and enhancement of EVLP-mediated reconditioning remains to be fully elucidated. In the current study, we demonstrate that EVs affect alveolar macrophages to decrease HR-induced HMGB1 and TNF-α secretion as well as diminishes iNKT cell-dependent IL-17A production. We have previously demonstrated that these immune cells play a critical role in the initiation and progression of lung IR injury as [3, 4, 25]. A significant upregulation of anti-inflammatory molecules (IL-10, KGF, PGE2) was observed after MSC and EVs treatment which further signifies the protective role of these treatment modalities. Previous studies have also suggested that elevated expression of KGF and PGE2 are a possible mechanism for the beneficial aspects of MSCs and EVs in models of lung injury and experimental sepsis, respectively [33, 34]. However, the effects of KGF are unclear in lung injury as a recent human clinical trial showed no improvement in physiological or clinical outcomes in ARDS patients that received recombinant KGF [35]. Our results show that one potential mechanism of EVs can be via paracrine secretion-dependent attenuation of specific immune cells i.e. macrophages and iNKT cell activation during lung IR injury. It is plausible that EVs may upregulate these paracrine factors, i.e. increased IL-10 expression, by modulating M2 macrophages or CD4 + CD25 + FoxP3+ T regulatory cell activation during lung IR.

Human MSC-derived EVs have been recently shown to attenuate acute lung injury when administered either intratracheally or intravenously in experimental studies [36–38]. The presence of human MSC and exosome-derived mitochondrial DNA in mouse lung tissue can be detected up to 28 days post injection after a single intratracheal or intravenous dose [38]. On the contrary, intratracheal administration of MSCs might be associated with rapid degradation and poor retention of the cells into the lung alveolar space, thereby impeding its complete biological effect [39]. These findings support the premise of our study focusing on EVs-derived from MSCs, rather than using MSCs themselves, as a therapeutic tool which can be administered safely via different routes thereby circumventing the issue of cell retention and viability. EVs comprise of both exosomes and microvesicles, either of which may contribute to the protective effects observed in lung IR injury, and are increasingly recognized as important mediators of cellular communication due to their capacity to merge with and transfer bioactive molecular contents to the recipient cells. Such factors include growth factors and their receptors, proteases, adhesion molecules, signaling molecules, as well as DNA, mRNA, miRNA and organelles (e.g. mitochondria) [40]. EVs have also been reported to shuttle specific patterns of miRNAs to modulate cell signaling and biological responses [29, 41, 42]. In addition, miRNAs have been implicated in dysregulation of gene expression in signaling pathways associated with IR injury and human lung transplantation [43–47]. In a recent study, Yu et al. demonstrated decreased cardiomyocyte apoptosis via translocation of miR-221, a potent anti-inflammatory miRNA, by MVs [48]. Furthermore, recent studies have demonstrated that miRNAs and MSCs can tightly regulate each other to alter the expression profile of key signaling pathways [48–52]. One such candidate is miR-206 which we have shown to be significantly upregulated in lung tissue after IR [53]. It is plausible that specific miRNAs contained within the EVs are crucial mediators of transcriptional regulation of inflammatory cytokine secretion of activated target immune cells like macrophages and iNKT cells in the context of lung IR injury. Future studies using specific antagomiRs (anti-sense miRNAs) and protectomiRs (overexpressing miRNAs) will help decipher the mechanistic pathways involved in EV-mediated protective effects via specific miRNAs in lung IR injury.

Previous studies from our group and others have shown that EVLP is a novel technique of normothermic lung perfusion using a “lung box” that allows for both the functional assessment of donor lungs as well as offering a platform for the rehabilitation of these lungs ex vivo prior to transplantation [12–14, 54–56]. However, this technology is in its infancy, and many questions remain regarding its potential application for the delivery of anti-inflammatory or immunomodulatory therapies, and its ability to rehabilitate marginal donor lungs [13, 15]. Recent reports demonstrate the potential of MSCs and EVs to restore alveolar fluid clearance in rejected donor lungs for transplantation [37, 57]. However, it is not well understood how they exert their protective phenotype and which specific lung cells are the targets of the paracrine or intercellular interactions involving EVs. Our results using the murine EVLP model demonstrate that EVs enhance EVLP-mediated protection for rehabilitation of DCD lungs by improving function and decreasing edema. Since endothelial barrier permeability is essential to maintain leukocyte trafficking and regulate pulmonary edema, our results indicate that EVs can effectively mitigate neutrophil transendothelial migration. This points to an important mechanism by which EVs can maintain endothelial barrier integrity and reduce edema in DCD lungs. These crucial findings demonstrate the ability of MSC-derived EVs as a viable therapeutic option to enhance the ability of EVLP-mediated reconditioning of functionally compromised DCD lungs for successful transplantation thereby offering a possibility of increasing lung donor pool size.

There are few limitations of the current study. First, the murine IR model recapitulates IR injury but does not involve murine lung transplantation. However, the acute lung injury and inflammation as well as the immunobiology observed in the hilar ligation model are consistent with biologic processes seen in murine and human lung transplant studies [58, 59]. Secondly, EVLP in the murine model was performed for 1 h compared to 4 h which is routinely performed in clinical EVLP protocols. Although 1 h of EVLP displayed significant improvements in the murine lungs, it is plausible that a longer time period of EVLP with repeated supplementation of EVs could result in better outcomes for the human lungs. This is specifically relevant for downregulation of gene expression and alteration of cytokine milieu from a proinflammatory profile to anti-inflammatory environment in the lungs during EVLP and subsequent transplantation.

## Conclusions

In summary, we demonstrated that MSC and EVs produce similar improvements to injured lungs during EVLP and after IR. A key aspect of EV-mediated protection is that not only involves direct modulation of immune cells such as macrophages and iNKT cells during IR, but also have the ability to protect against increased microvascular permeability and resultant edema during EVLP. Since innate immunity has been implicated in the development of acute allograft dysfunction in human lung transplantation, we show that EVs are MSC derivatives that can be effectively used in the immunomodulation of lung inflammation after IR. We further propose that EVs can be an effective supplement for Steen solution using the EVLP platform to deliver these biologically active MSC-derivatives in the rehabilitation of marginal donor lungs for successful transplantation. Future studies using modified EVs employing novel strategic molecules i.e. miRNAs can offer possible therapeutic avenues to further explore the clinical translation of these cell-free secretory vesicles in the regeneration of tissue damage in patients undergoing lung transplantation.

## Abbreviations

DCD: Donation after circulatory death
EV: Extracellular vesicles
EVLP: ex vivo lung perfusion
HMGB1: High mobility group box 1
HR: Hypoxia/reoxygenation
IR: Ischemia-reperfusion
MSC: Mesenchymal stromal cells
